# Characterization and Insecticidal Efficacy of Green-Synthesized Silver Nanoparticles Against Four Stored Product Insect Species

**DOI:** 10.3390/insects17020143

**Published:** 2026-01-27

**Authors:** Daniel Martínez-Cisterna, Olga Rubilar, Leonardo Bardehle, Manuel Chacón-Fuentes, Lingyun Chen, Benjamin Silva, Marcelo Lizama, Pablo Parra, Ignacio Matamala, Orlando Barra, Ramón Rebolledo

**Affiliations:** 1 Centro de Investigación Biotecnológica Aplicada al Medio Ambiente (CIBAMA), Universidad de La Frontera, Av. Francisco Salazar 01145, Temuco 4811230, Chile; 2 Laboratorio de Entomología Aplicada, Facultad de Ciencias Agropecuarias y Medioambiente, Universidad de La Frontera, Av. Francisco Salazar 01145, Temuco 4811230, Chile; 3 Department of Chemical Engineering, Universidad de La Frontera, Av. Francisco Salazar 01145, Casilla 54-D, Temuco 4811230, Chile; 4 Departamento de Producción Agropecuaria, Facultad de Ciencias Agropecuarias y Medioambiente, Universidad de La Frontera, Av. Francisco Salazar 01145, Temuco 4811230, Chile; 5 Agriaquaculture Nutritional Genomic Center (CGNA), Temuco 4780000, Chile; 6 Department of Agriculture, Food and Nutritional Sciences, University of Alberta, Edmonton, AB T6G 2P5, Canada; 7 Programa de Magíster en Ciencias Mención Genética, Facultad de Ciencias, Universidad Austral de Chile, Av. Rector Eduardo Morales Miranda 23, Valdivia 5090000, Chile; 8 Programa de Doctorado en Ciencias Agroalimentarias y Medioambiente, Facultad de Ciencias Agropecuarias y Medioambiente, Universidad de La Frontera, Av. Francisco Salazar 01145, Temuco 4811230, Chile; 9 Programa de Doctorado en Ciencias de Recursos Naturales, Universidad de La Frontera, Av. Francisco Salazar 01145, Temuco 4811230, Chile

**Keywords:** green synthesis, sustainable pest management, wheat flour, corn kernels, nanotechnology

## Abstract

This study aimed to biosynthesize silver nanoparticles (AgNPs) using aqueous leaf extract of *Galega officinalis* and to evaluate their insecticidal activity against key stored-product pests. AgNP formation was confirmed through UV–vis spectroscopy, which showed a surface plasmon resonance peak at 380 nm. FTIR analysis indicated the presence of plant-derived functional groups likely involved in the reduction and stabilization of Ag^+^ ions. Dynamic light scattering revealed an average hydrodynamic diameter of 25.07 nm, a PDI of 0.39, and a zeta potential of −22 mV, while TEM images showed predominantly spherical and polydisperse particles ranging from 4.3 to 42.4 nm. Insecticidal bioassays performed on *Sitophilus granarius*, *Tribolium confusum*, *Plodia interpunctella*, and *Ephestia kuehniella* revealed concentration-dependent mortality. The highest mortality rates were recorded at 1000 ppm, reaching 100% in *T. confusum*, 83.33% in *P. interpunctella*, and 76.67% in both *S. granarius* and *E. kuehniella*. These findings demonstrate the potent insecticidal activity of *G. officinalis*-mediated AgNPs and support their potential as environmentally friendly alternatives for stored-product pest management, warranting further studies on safety, large-scale synthesis, and integration into pest-control programs.

## Simple Summary

This study describes the green synthesis of silver nanoparticles (AgNPs) using *Galega officinalis* leaf extract and their insecticidal effects against four stored-product pests: *Sitophilus granarius*, *Tribolium confusum* (adults), *Plodia interpunctella*, and *Ephestia kuehniella* (larvae). AgNPs were characterized by UV–Vis, FTIR, DLS, and TEM. Bioassays using AgNPs (10–1000 ppm) mixed in flour and diet showed dose-dependent mortality after 7 and 31 days, respectively. The highest susceptibility was observed in *T. confusum* and *E. kuehniella*. These results suggest the potential of *G. officinalis*-based AgNPs as eco-friendly candidates for stored pest control.

## 1. Introduction

Pests represent a major constraint to global food production and agricultural productivity [1]. Herbivorous insects can cause severe yield losses, exceeding 80% under intense infestations [2]. During the postharvest stage, particularly in stored food products, effective pest management is essential to preserve both quality and safety [3].

Among the most destructive storage pests are the red flour beetle (*Tribolium confusum* Jacquelin du Val, 1968; Coleoptera: Tenebrionidae) and the granary weevil (*Sitophilus granarius* Linnaeus, 1758; Coleoptera: Curculionidae). *T. confusum* primarily infests milled grains and flours, releasing pheromones and toxic benzoquinones that impart off-odors and unpleasant flavors, thus reducing product quality [4,5]. Its larvae feed on damaged grains, further decreasing their market value. Conversely, *S. granarius* lays its eggs inside intact grains, where larvae develop internally, consuming the endosperm or embryo. In addition to direct damage, infestations by these beetles facilitate moisture migration and localized heating, creating conditions favorable to mold proliferation and mycotoxin production [6,7].

Other common pests of stored cereals include the Indian meal moth (*Plodia interpunctella* Hübner, 1813; Lepidoptera: Pyralidae), and the Mediterranean flour moth (*Ephestia kuehniella* Zeller, 1879; Lepidoptera: Pyralidae), both of which infest a wide range of cereal-based products and other dry goods [8,9]. Their larvae contaminate flour with silk webbing, exuviae, and frass, often rendering it unfit for commercialization [10].

Historically, chemical insecticides such as phosphine, organophosphates, and pyrethroids have been extensively used to control these pests. However, their use is increasingly restricted due to concerns over environmental pollution, resistance development, toxicity to non-target organisms, and risks to human health [11,12,13,14].

In response, nanotechnology has emerged as a promising and sustainable tool for pest management. Among various nanomaterials, silver nanoparticles (AgNPs) have received special attention due to their broad-spectrum antimicrobial and insecticidal properties. These effects involve multiple mechanisms, including membrane disruption, oxidative stress, and interference with respiration and development [15,16,17].

Recently, green synthesis of AgNPs using plant extracts has gained interest as an eco-friendly approach that avoids toxic reagents and reduces production costs. This method leverages phytochemicals as both reducing and stabilizing agents, resulting in stable and bioactive nanoparticles [18,19,20,21]

In this context, *Galega officinalis* L. (commonly known as goat’s rue), a leguminous plant native to Europe and introduced in South America, has attracted interest due to its rich phytochemical profile, which includes flavonoids, alkaloids, and saponins with known antimicrobial and medicinal properties [22]. Notably, *G. officinalis* extract has already been employed to biosynthesize AgNPs, yielding polydisperse nanostructures with demonstrated antibacterial and insecticidal activities [23].

Building upon this background, the present study aimed to synthesize AgNPs using aqueous extracts of *G. officinalis* leaves and evaluate their insecticidal efficacy under laboratory conditions against four major stored-product pests: *S. granarius*, *T. confusum*, *P. interpunctella*, and *E. kuehniella*.

## 2. Materials and Methods

### 2.1. Plant Collection

Leaves of *Galega officinalis* were collected from rural areas surrounding the Maquehue sector (Temuco, La Araucanía Region, Chile). The plant material was thoroughly rinsed several times with potable water to remove particulate matter and surface impurities. Subsequently, the leaves were air-dried under controlled laboratory conditions at 25 °C for 24 h to reduce moisture content prior to further processing.

### 2.2. Biosynthesis of Silver Nanoparticles

Ten grams of *Galega officinalis* leaves were placed in a 250 mL Erlenmeyer flask containing 100 mL of deionized water and boiled at 100 °C for 5 min. The resulting aqueous extract was allowed to cool to room temperature (25 ± 2 °C) and filtered under vacuum using Whatman No. 1 filter paper.

The biosynthesis of silver nanoparticles (AgNPs) was carried out following the methodology described by Manosalva et al. [23]. Briefly, the leaf extract was mixed with a 1.5 mM solution of silver nitrate (AgNO_3_), and the pH of the mixture was adjusted to 11. The synthesis reaction was maintained at room temperature (25 ± 2 °C) under continuous magnetic stirring for 24 h.

### 2.3. Characterization of Biosynthesized Silver Nanoparticles

The bioreduction of Ag^+^ ions was monitored via UV-Vis spectroscopy (Genesys10S spectrophotometer, Thermo Fisher Scientific, Madison, WI, USA) by recording absorption spectra in the range of 200–800 nm.

The average hydrodynamic diameter (nm), polydispersity index (PDI), and zeta potential (mV) of the biosynthesized AgNPs were measured using a Zetasizer Nano Zs (Malvern Instruments Co., Ltd., Malvern, UK). Measurements were conducted at 25 °C using disposable folded capillary zeta cells (10 mm path length) and a fixed detection angle of 173°. Prior to analysis, samples were sonicated to ensure homogeneous dispersion.

Nanoparticle morphology and size were analyzed by Transmission Electron Microscopy (TEM) using a Talos F200C G2 instrument (Thermo Fisher Scientific, Hillsboro, OR, USA) equipped with a Ceta 16M CMOS Camera (pixel size: 14 μm × 14 μm; 16 bit resolution).

### 2.4. Insects Culture

Specimens of *Sitophilus granarius*, *Tribolium confusum*, *Plodia interpunctella*, and *Ephestia kuehniella* were obtained from long-established laboratory colonies maintained at the Laboratorio of Applied Entomology, Universidad de La Frontera (Temuco, Chile). Species identification was based on morphological traits using the taxonomic keys provided by Artigas [24].

Adults of *S. granarius* and *T. confusum* were used in bioassays, whereas third-instar larvae were selected for *P. interpunctella* and *E. kuehniella*. Each species was maintained on species-specific diet: whole corn kernels for *S. granarius*, wheat flour for *T. confusum*, and a mixture of wheat flour with walnut (*Juglans regia*) for the lepidopteran species. Insects were reared in 6 L plastic containers filled to one-third with the respective diet, which was replenished periodically. All cultures were kept under controlled conditions (22 ± 1 °C, 40 ± 5% relative humidity, and a 12:12 h light:dark photoperiod).

### 2.5. Insecticidal Effect of Biosynthesized AgNPs

The insecticidal effect of AgNPs on *S. granarius* and *T. confusum* was evaluated using 50 mg of corn kernel powder and 50 mg of wheat flour, respectively, as dietary substrates, placed in 100 mm Petri dishes. Each dish was treated with 150 µL of one of four AgNP concentrations (10, 250, 500 y 1000 ppm). Five adults of the corresponding species were introduced per dish, followed by an additional 150 µL of the same AgNP solution applied directly onto the insects.

Each concentration was tested with 15 replicates, each containing five insects (*n* = 450 in total). Deionized water and AgNO_3_ (1.5 mM) were used as negative and positive controls, respectively. Mortality was recorded every 24 h for seven days. Percentage mortality was calculated using Abbot’s [25] formula:

(1) $$Percentage\;of\;mortality=\frac{Number\;of\;dead\;individuals}{Number\;of\;treated\;individuals}\times 100$$

For *P. interpunctella* and *E. kuehniella*, the bioassay used 50 mg of wheat flour per Petri dish, treated with the same concentrations and volumes of AgNPs. Two third-instar larvae were placed in each Petri dish, and 150 µL of AgNP solution was applied directly to the larvae.

Each concentration was evaluated with 15 replicates (*n* = 180 total. Negative and positive controls were the same. Mortality was recorded every 24 h for 31 days, covering the full developmental cycle.

All experiments were performed under standardized conditions (24 ± 1 °C, 40 ± 5% relative humidity, and a 12:12 h light:dark photoperiod).

### 2.6. Statistical Analysis

Mortality data were analyzed using IBM SPSS statistics v20 (IBM corp., Armonk, NY, USA). One-way ANOVA followed by Tukey’s HSD test was used to determine significant differences among treatment groups (*p* ≤ 0.05). Results are reported as mean ± standard error (SE), and different letters indicate statistically significant differences according to Tukey’s test.

## 3. Results and Discussion

### 3.1. Characterization of AgNPs

The biosynthesis of silver nanoparticles (AgNPs) was evidenced by a visible color change from yellow to dark brown upon the addition of 1.5 mM AgNO_3_ (adjusted to pH 11) to the aqueous leaf extract of *Galega officinalis*. This chromatic shift is indicative of silver ion reduction (Ag^+^ → Ag^0^) and the subsequent formation of colloidal nanoparticles [26]. The observed phenomenon is commonly attributed to the excitation of surface plasmon resonance (SPR), a distinctive optical property of AgNPs [27].

#### 3.1.1. UV-Vis Spectroscopy

**Figure 1:**
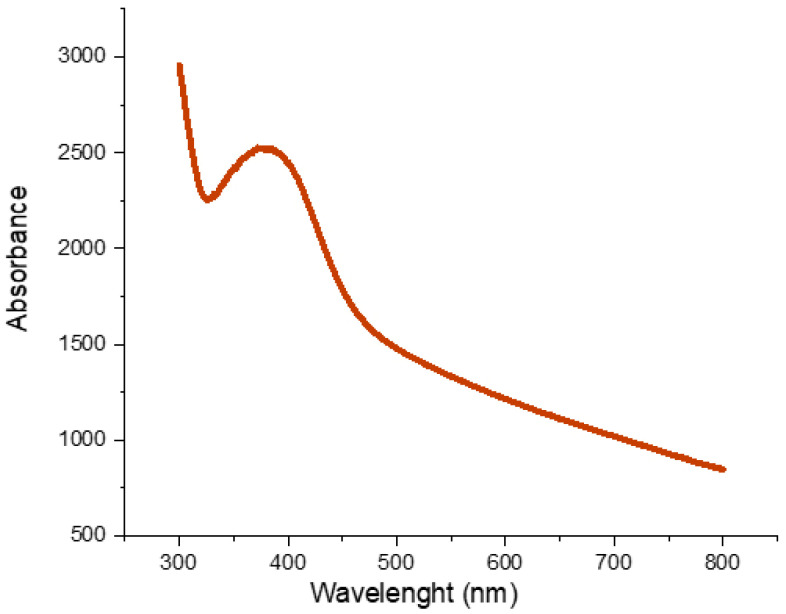
UV-Vis absorption spectrum of biosynthesized AgNPs.

The surface plasmon resonance (SPR) of AgNPs was confirmed by UV-visible spectroscopy, showing a maximum absorption peak at 380 nm (Figure 1). This peak corresponds to the collective oscillation of conduction band electrons on the nanoparticle surface in response to incident light, confirming the reduction of Ag^+^ to metallic silver (Ag^0^) [28]. Similar SPR peaks have been reported in other plant-mediated syntheses, such as those using *Acacia nilotica* extract [29], supporting the reliability of the biosynthetic approach.

#### 3.1.2. FTIR Analysis

**Figure 2:**
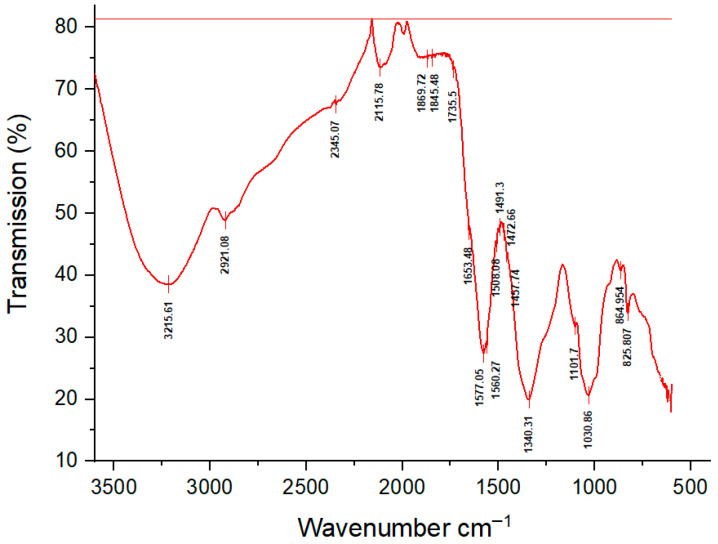
FTIR spectrum of biosynthesized AgNPs.

Fourier-transform infrared spectroscopy (FTIR) was conducted to identify the functional groups involved in the reduction and stabilization of AgNPs synthesized with *G. officinalis* leaf extract. The FTIR spectrum (Figure 2) exhibited prominent absorption bands at 3215.61 cm^−1^ and 2921.08 cm^−1^, corresponding to O-H stretching vibrations of hydrogen-bonded phenolic compounds and =C-H stretching of alkenes, respectively [29,30,31,32]. A strong peak at 2345.07 cm^−1^ was attributed to aliphatic cyanide/nitrile groups [33].

Additional bands at 2115.78 cm^−1^ and 1735.5 cm^−1^ were assigned to C=C stretching vibrations of alkynes and C=O stretching of esters and carboxylic acids, respectively [34]. The peak observed at 1577 cm^−1^ likely resulted from C=C stretching of aromatic rings [35]. A strong band at 1340.31 cm^−1^ was associated with C-N stretching of aliphatic amines [36], while the peak at 1030.86 cm^−1^ corresponded to C-O stretching vibrations of alcohols [1]. Peaks between 864.95 cm^−1^ and 825.81 cm^−1^ were indicative of C-H bending in alkenes and aromatic moieties [36].

These results highlight the presence of multiple functional groups, including hydroxyl, alkenes, alkyne, amine, ester, and carboxylic acid groups, which are known to participate in the bioreduction and capping of AgNPs. These functional moieties are likely derived from proteins and other bioactive constituents in the plant extract [37,38]. This is consistent with previous findings that phytochemicals such as terpenoids, reducing sugars, flavonoids, and alkaloids play a pivotal role in the green synthesis of AgNPs [39].

#### 3.1.3. Hydrodynamic Diameter, PDI and Zeta Potential

**Figure 3:**
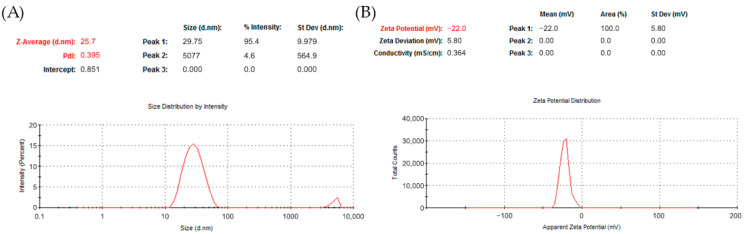
Dynamic light scattering (DLS) and zeta potential analysis of biosynthesized AgNPs. (**A**) Size distribution by intensity showing a Z-average diameter of 25.07 nm and a polydispersity index (PDI) of 0.39. (**B**) Zeta potential distribution with a mean value of −22 mV, indicating moderate colloidal stability.

Dynamic light scattering (DLS) analysis revealed that the biosynthesized AgNPs exhibited an average hydrodynamic diameter of 25.07 nm, with a polydispersity index (PDI) of 0.39 (Figure 3A). This PDI value indicates a moderate degree of size distribution heterogeneity among the nanoparticles. The corresponding zeta potential was measured at −22 mV (Figure 3B), suggesting moderate electrostatic stability due to repulsive forces between negatively charged particles in suspension.

The DLS profile showed a single dominant peak, indicating that most particles were distributed within a relatively narrow size range. However, the observed PDI confirms the presence of polydispersity, which is further supported by the zeta potential value. Particles with zeta potentials more negative than −30 mV are generally considered highly stable; thus, the measured value indicates moderate colloidal stability in aqueous media.

These observations are in agreement with the TEM results described in Section 3.1.4, which showed that the AgNPs had a size range of approximately 4.3 to 42.4 nm. The combination of DLS and zeta potential data confirms that the synthesized AgNPs are moderately polydisperse and electrostatically stable, supporting their suitability for biological applications [40].

#### 3.1.4. TEM Analysis

**Figure 4:**
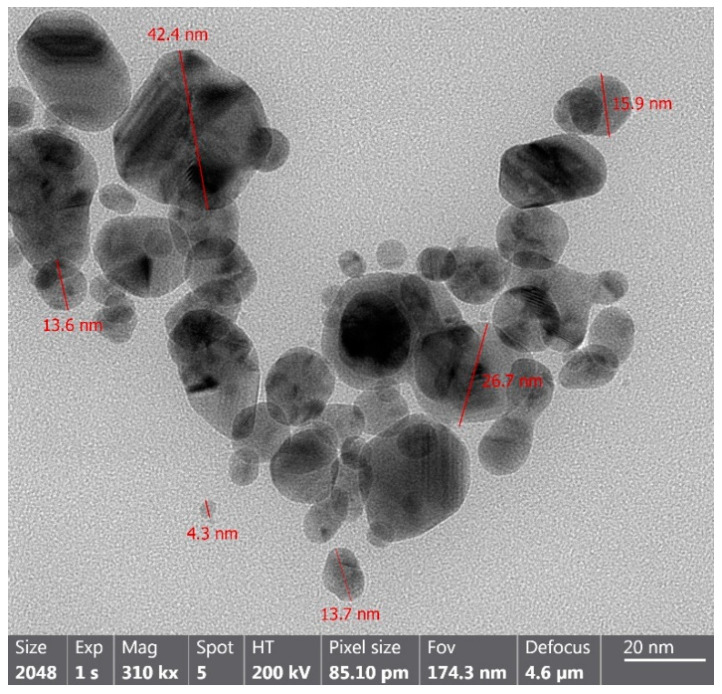
TEM pictures of AgNPs at 310 kx magnification. The image revealed sizes ranging from 4.3 to 42.4 nm showing the morphological characteristics of biosynthesized AgNPs.

Transmission electron microscopy (TEM) analysis confirmed the formation of polydisperse AgNPs with predominantly spherical morphology and particle sizes ranging from 4.3 to 42.4 nm (Figure 4). These findings are consistent with the DLS results (Section 3.1.3), which also indicated a moderate degree of polydispersity.

Similar morphological patterns have been reported in previous studies employing plant-mediated synthesis. For example, Seekonda and Rani [41] described AgNPs with irregular spherical shapes using *Embelia robusta* extract, while Thirunavoukkarasu et al. [42] reported AgNPs ranging from 8 to 90 nm with *Bougainvillea glabra*. Sankar et al. [43] obtained spherical particles of 5–25 nm from *Avicennia marina*, and Cheng et al. [44] reported diameters between 27.8 and 36.5 nm using *Flos sophorae* extract.

### 3.2. Insecticidal Effect of Biosynthesized AgNPs

#### 3.2.1. Insecticidal Effect of Biosynthesized AgNPs on Coleoptera

**Figure 5:**
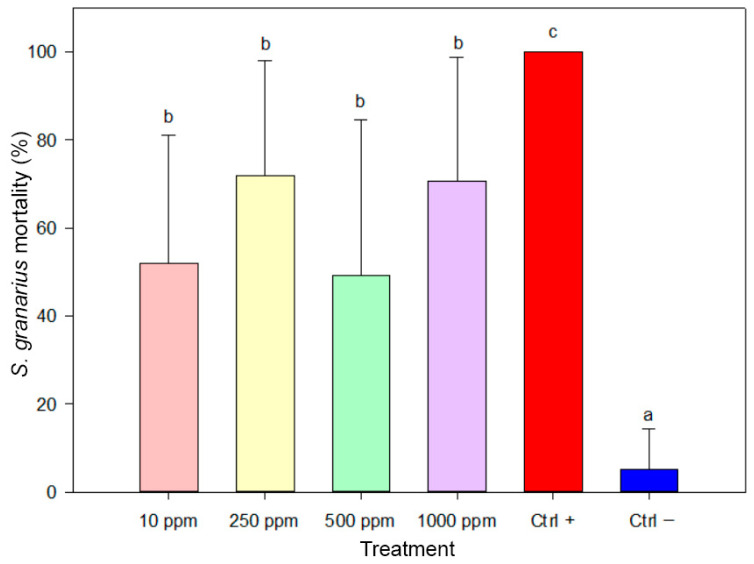
Mortality of *Sitophilus granarius* adults after seven days of exposure to biosynthesized AgNPs. Bar represents mortality values (±SE). Treatments sharing the same letter are not significantly different (ANOVA, Duncan’s HSD test, *p* ≥ 0.05).

The biosynthesized AgNPs demonstrated significant insecticidal activity against *Sitophilus granarius* adults (Figure 5). The highest mortality rates were recorded at 1000 ppm (76.67%) and 250 ppm (72.00), closely approaching the 100% mortality observed in the positive control group. In contrast, lower but substantial mortality was observed at 10 ppm (52.00%) and 500 ppm (49.33%).

Despite the differences in mortality percentages across concentrations, no statistically significant differences were detected among the AgNP-treated groups. However, all concentrations resulted in significantly higher mortality compared to the negative control (15.00%). These differences were statistically significant according to one-way ANOVA (F(5,84) = 24.711, *p* < 0.001)

**Figure 6:**
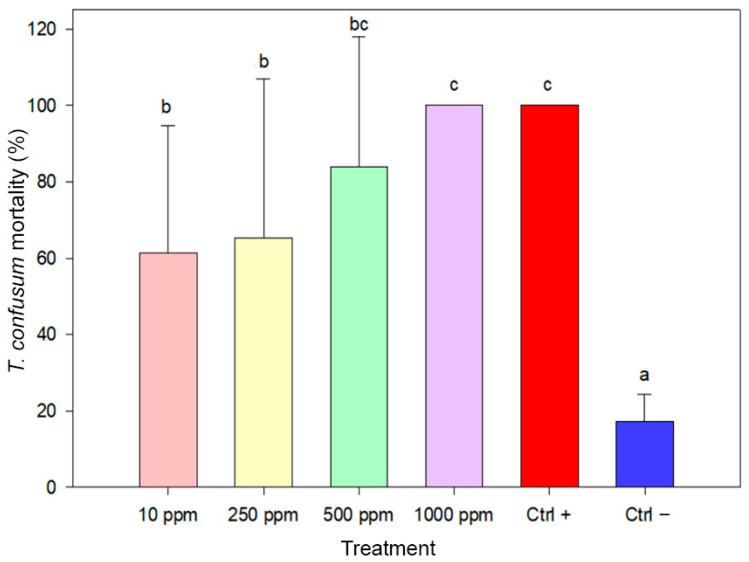
Mortality of *Tribolium confusum* adults after seven days of exposure to biosynthesized AgNPs. Bars represent mean mortality values (±SE). Treatments sharing the same letter are not significantly different (ANOVA, Duncan’s HSD test, *p* ≥ 0.05).

Biosynthesized AgNPs exhibited strong insecticidal activity against adults of *T. confusum* (Figure 6). The highest mortality rate was recorded at 1000 ppm, reaching 100%, a value equivalent to the positive control. This was significantly different from the lowest concentration (10 ppm), which produced 61.33% mortality, and from the negative control (17.33%). Intermediate mortality levels were recorded at 250 and 500 ppm, with values of 65.33% and 84.00%, respectively. All AgNP treatments differed significantly from the negative control, confirming their high insecticidal efficacy. One-way ANOVA indicated statistically significant differences among treatments (F(5,84) = 21.544, *p* < 0.001)

These results are consistent with previous studies evaluating the insecticidal properties of AgNPs against stored-product beetles. For instance, Eyssa et al. [45] reported 100% mortality in *S. granarius* using high-density polyethylene (HDPE) incorporated with synthetic AgNPs. Similarly, Sankar et al. [43] achieved 80% and 100% mortality in *S. oryzae* at concentrations of 100 and 25 ppm, respectively, employing AgNPs biosynthesized from *Avicinia marina*. In another study, Almadiy et al. [46] determined LC_50_ values between 4.1 and 11.4 µg/cm^2^ for larvae and adults of *Trogoderma granarium* treated with AgNPs derived from *Peganum harmala* alkaloids.

The high susceptibility observed in coleopteran species may be attributed to multiple toxicological mechanisms triggered by AgNPs, including disruption of gut epithelial, respiratory inhibition, and oxidative stress due to the generation of reactive oxygen species (ROS), all of which have been widely reported in insects [47].

#### 3.2.2. Insecticidal Effect of Biosynthesized AgNPs on Lepidoptera

**Figure 7:**
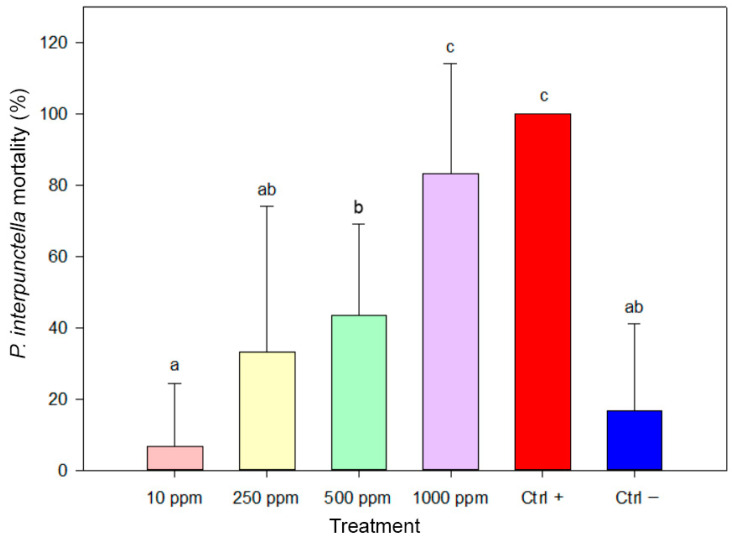
Mortality of *Plodia interpunctella* larvae after 30 days of exposure to biosynthesized AgNPs. Bars represent mean mortality values (±SE). Treatments sharing the same letter are not significantly different (ANOVA, Duncan’s HSD test, *p* ≥ 0.05).

Biosynthesized AgNPs demonstrated significant insecticidal activity against larvae of *P. interpunctella* (Figure 7). The lowest mortality rate (6.67%) was recorded at 10 ppm, showing no significant difference from the negative control. Mortality increased with concentration: 33.33% at 250 ppm and 43.33% at 500 ppm, although these values did not differ significantly from each other. In contrast, the highest mortality rate was observed at 1000 ppm (83.33%), which did not differ significantly from the positive control, but was statistically higher than all other AgNP treatments. One-way ANOVA confirmed significant differences among treatments (F(5,84) = 29.535, *p* < 0.001).

**Figure 8:**
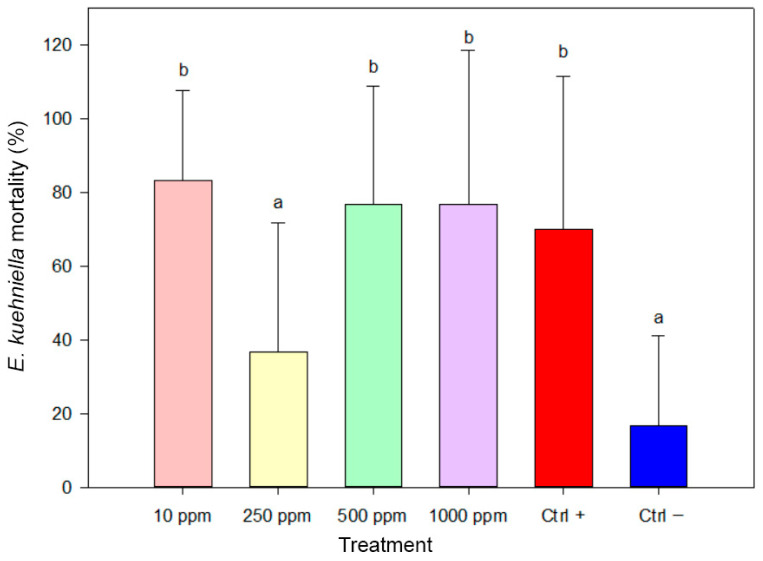
Mortality of *Ephestia kuehniella* larvae after 30 days of exposure to biosynthesized AgNPs. Bars represent mean mortality values (±SE). Treatments sharing the same letter are not significantly different (ANOVA, Duncan’s HSD test, *p* ≥ 0.05).

Similarly, biosynthesized AgNPs were effective against *Ephestia kuehniella* larvae (Figure 8). The highest mortality rate (83.33%) was unexpectedly observed at the lowest tested concentration (10 ppm), showing no significant difference from the positive control (70.00%). Treatments at 500 ppm and 1000 ppm also showed high efficacy, with mortality rates of 76.67%, both statistically comparable to positive control. In contrast, 250 ppm resulted in only 36.67% mortality, which was not significantly different from the negative control. These results suggest a non-linear dose–response in *E. kuehniella*, possibly due to behavioral or physiological variations. One-way ANOVA confirmed significant differences among treatments (F(5,84) = 9.443, *p* < 0.001).

These findings align with other reports of AgNP toxicity in lepidopteran pests. For example, *Plutella xylostella* exhibited an LC_50_ of 0.691 mg/mL [16], while *Spodopttera litura* showed LC_50_ values ranging from 0.0312 mg/L to –46.9 mg/L). In *Earias vittela*, LC_50_ values ranged from 25.12 mg/L to 45.46 mg/L, and in *Bombyx mori*, 100% mortality was observed at 1600–3200 mg/L. Likewise, *Agrotis ipsilon* and *Trichoplusia ni* showed LC_50_ values of 5.20 mg/mL and 0.81 mg/mL, respectively, with larvae being the most susceptible stage [26,31,48,49,50,51,52]. Manimegalai et al. [51] further reported strong antifeedant effects of AgNPs on *S. litura* (78.77%) and *Helicoverpa armigera* (82.16%), with maximum larval mortality rates of 78.77% and 72.70% at 150 mg/L.

The observed insecticidal effects may be attributed to the synergistic action of AgNPs and plant-derived phytochemicals from *G. officinalis,* such as flavonoids, alkaloids and phenolics. These compounds, acting as surface capping agents, may enhance toxicity through mechanisms including feeding deterrence, disruption of midgut epithelium, and interference with hormonal regulation of larval development [26,52].

Overall, the effectiveness of AgNPs depends on various physicochemical parameters such as concentration, particle size, surface functionalization, crystallinity, zeta potential, and aggregation state, which collectively influence their interaction with biological systems and capacity to induce oxidative stress and cellular damage [52].

## 4. Conclusions

The present study demonstrated that *G. officinalis* aqueous leaf extract can be effectively employed for the green synthesis of silver nanoparticles (AgNPs), yielding spherical and polydisperse particles with sizes ranging from 4.3 to 42.4 nm. Spectroscopic and microscopic analyses confirmed the presence of plant-derived biomolecules responsible for the reduction and stabilization of AgNPs, as well as their moderate colloidal stability. Bioassays against four major stored-grain pests, *S. granarius*, *T. confusum*, *P. interpunctella*, and *E. kuehniella*, revealed concentration-dependent insecticidal activity. Notably, *T. confusum* and *E. kuehniella* showed the highest susceptibility, followed by *S. granarius* and *P. interpunctella*. These results are consistent with previous studies on the insecticidal efficacy of biosynthesized AgNPs and highlight their potential as alternative control agents within integrated pest management programs. Nevertheless, future research should focus on upscaling synthesis, evaluating effects on non-target organisms, and ensuring environmental safety prior to practical application.

## Data Availability

The original contributions presented in this study are included in the article. Further inquiries can be directed to the corresponding authors.
